# Supplementary material for: Are carbon emissions trading and green financial instruments synergistic? -Comprehensive quantitative research based on content analysis
Source: PLoS One. 2024 Mar 7;19(3):e0298601. doi: 10.1371/journal.pone.0298601 (PMC10919601; doi:10.1371/journal.pone.0298601)
Supplement: S1 Data — (ZIP) [file pone.0298601.s001.zip › Data/地级市Z_F工作报告.zip!/╕ú╜¿/─■╡┬╩╨/└┤╫╘╣1⁄2╓┌║┼í╛┬φ┐╦╩2╛▌═°í┐_2014.docx]

各位代表：

现在，我代表宁德市人民政府向大会报告政府工作，请予审议，并请市政协各位委员和其他列席的同志提出意见。

一、2013年工作回顾

过去的一年，在省委、省政府和市委的正确领导下，我市各级政府坚持以邓小平理论、“三个代表”重要思想、科学发展观为指导，认真学习贯彻党的十八大、十八届三中全会和习近平总书记系列重要讲话精神，把握“稳中求进”的总基调，全力推进科学发展、加快发展，较好地完成了市三届人大二次会议确定的年度主要目标任务，实现了“六新大宁德”建设良好开局。

初步统计，2013年全市生产总值1230亿元、增长12.6%；公共财政总收入126亿元、增长20.4%，地方公共财政收入89亿元、增长25.6%；全社会固定资产投资934亿元、增长47%；出口总值28.38亿美元、增长29.6%；实际利用外资1.44亿美元、增长20.2%；社会消费品零售总额370亿元、增长15.4%；居民消费价格总水平控制在2.2%；城镇居民人均可支配收入24008元、增长10%；农民人均纯收入10065元、增长14%。

（一）产业发展水平提升

现代农业增产增效。加大强农惠农富农力度，出台加快现代农业发展的一系列政策措施，全市农林牧渔业总产值386亿元、增长5.9%，粮食总产量稳定在66万吨。8个省级农民创业园、示范基地和11个市级高优农业示范园加快建设，新增设施农业面积5000亩。新获批国家和省级农业标准化示范区9个，新增省级农业龙头企业22家、农民专业合作示范社22家、国家地理标志证明商标5件，福安市荣获“中国油茶之乡”称号，宁德大黄鱼、霞浦海参入选全省十大渔业品牌。农业基础设施进一步完善，开发整理土地6.8万亩，除险加固水库44座、海堤14条，解决59.53万农村居民饮水安全问题。农业“五新”深入推广，农机、气象、水文等服务保障有力，重大自然灾害应急管理协调机制进一步健全，有效应对“菲特”等强台风和暴雨袭击，最大限度减少了灾害损失。

工业经济稳定增长。着力“抓龙头、铸链条、建集群”，出台促进工业稳定增长的一系列政策措施，完成技改投资149亿元，全市规模以上工业总产值2313亿元、增加值564亿元，分别增长21.6%和19%，形成6个产值超百亿产业集群，新增产值亿元以上企业106家、省级战略性新兴产业企业120家、中国驰名商标7件。加大中小企业扶持力度，在全省率先开展“助保贷”、“成长贷”业务，发行中小企业集优票据2.8亿元。柘荣经济开发区获批省级开发区，霞浦台湾水产品集散中心被列为首批省级海洋产业示范园区，全市工业园区完成投资237亿元，实现规模以上工业产值1479亿元。

第三产业不断壮大。全年实现增加值398亿元、增长8.5%。新增限额以上商贸流通企业169家，一批城市商业综合体开工建设。旅游业加快发展，成功举办第三届宁德世界地质公园文化旅游节，太姥山、九龙漈·鲤鱼溪分别被评定为国家5A级、4A级景区，嵛山岛、台山岛、三都岛入选福建省“十大美丽海岛”，全市共接待游客1346万人次，旅游收入103亿元，分别增长21.4%和22.8%。金融服务保障有力，村镇银行组建全面启动，中信银行、厦门国际银行宁德分行开业，年末金融机构本外币贷款余额1172亿元、增长15.2%。

（二）项目带动支撑有力

“五大战役”完成年度计划127.2%，在建重点项目完成投资502亿元、占年度计划146.3%，“双百项目”完成投资430亿元、占年度计划139.3%。宁德核电站1号机组、鼎信镍业一期、新能源科技二期、益联远大一期等一批重大产业项目建成投产，鼎信科技、中聚天冠燃料乙醇项目动工建设。衢宁铁路获批立项，漳湾8号、9号码头主体工程完工，一批高速公路、铁路、普通公路和港航项目加快建设。市图书馆、妇女儿童活动中心、老年人体育活动中心、市医院新院区一期工程等一批民生项目建成。招商选资持续深化，全年签约“三维”项目254个、总投资1529亿元。

（三）城乡发展协调推进

新一轮城市总体规划深入实施，中心城市实施建设项目143个、完成投资86亿元。着力解决群众关切问题，完成单石碑垃圾堆放场改造、建筑垃圾填埋场建设和闽东中路拆迁工程，收回南漈公园管理权。薛令之路建成通车，第三水厂续建工程、贵岐山污水处理厂二期工程建成投产。进一步理顺城市管理行政执法体制，环境卫生、违规广告、建筑渣土整治成效明显，中心城区智能公交推广走在全省前列，省级卫生城市创建工作扎实推进。县域经济竞相发展，综合实力持续增强，福鼎市再次荣获全省县域经济发展“十佳”称号，古田县经济发展指数由全省第49位提升到第23位。小城镇综合改革建设试点迈出新步伐，新增南阳、大甲2个参照省级试点镇和9个市级试点镇，全年完成小城镇建设投资157亿元。18个新农村试点示范村建设取得明显成效。

（四）改革开放持续深化

农村综合改革扎实推进，集体土地确权发证率98.7%，农民承包地流转率16.5%，森林综合保险参保率93%。行政审批制度改革取得实效，市级审批事项削减到184项，精简率达44.4%，即办率达80%以上。改进国有资产监管方式和企业经营管理，市属国有企业资产总额达228亿元、增长16.3%。对外开放持续拓展，新批外商投资、增资项目32项，白马港鼎信码头外贸作业点通过省级验收，沙埕口岸启动外贸业务。闽东北经济协作区重点协作项目完成投资292亿元。宁台交流合作不断深化，联德冶金新材料项目加快建设，台湾科技产业园落户福安市，三沙对台小额贸易口岸获批试行更开放管理措施，第四届海峡两岸电博会、第五届海峡论坛·陈靖姑文化节和海峡两岸各民族欢度“三月三”节成功举办。

（五）社会建设不断进步

民生保障持续改善。全年公共财政民生支出121亿元、增长25.3%，36件为民办实事项目基本完成。出台扶持高校毕业生自主创业政策措施，加强就业服务和用工保障，城镇新增就业3.01万人，农村劳动力转移就业4.09万人，城镇登记失业率1.82%。城乡医疗保险实现全覆盖。居民社会养老保险制度实现城乡一体化，参保率96%。城乡低保标准和补助水平进一步提高，全年共发放低保金2.64亿元、增长15%。新开工保障性住房8957套，基本建成8716套，历年配租配售率达96.5%。市社会福利中心和福乐家园主体工程完成，建成各类社会福利机构266个。扶贫开发迈出新步伐，出台配套政策进一步支持6个省级扶贫开发重点县加快发展，9个县（市、区）全部纳入比照享受原中央苏区政策范围。完成“造福工程”危房改造2.3万人。新建和改造农村公路474公里，提前一年半完成农村公路安保工程，全市建制村基本实现通客车。

社会事业协调发展。实施校安工程32项、城区中小学扩容工程16项，新改扩建公办幼儿园38所，东侨开发区与福安市、福鼎市分别通过义务教育发展基本均衡县（市、区）国家和省级验收，宁德师院通过学士学位授予权评估。实施省级以上科技项目62项，获全省科技进步奖10项，引进各类急需紧缺人才206人，宁德国家农业科技园区获批设立。新建群众性文化激情广场示范点40个，新增全国重点文物保护单位6个。全民健身活动广泛开展，建成城市社区多功能运动场21个，成功举办第四届市运会，宁德籍运动员在第十二届全运会荣获3枚金牌。新增医疗机构床位510张，“海云工程”覆盖全市所有乡镇卫生院、社区卫生服务中心和516个村卫生所，周宁县医院被列为全国第二批公立医院改革试点，柘荣县被授予“中国长寿之乡”称号。完成人口计生工作责任书任务，人口自然增长率5.9‰。妇女、儿童、老龄和残疾人工作得到加强。

社会保持和谐稳定。深入推广“霞浦经验”，持续开展领导干部接访、下访，信访和社会矛盾多元调解工作不断加强，矛盾纠纷调处成功率98.1%，按“路线图”依法处理信访事项答复率100%。严厉打击违法犯罪，群众对社会治安满意率达93.6%。食品药品安全监管不断强化。道路交通、消防、建筑施工等重点领域整治深入开展，全市安全生产形势保持稳定。落实大学生士兵优待政策，双拥共建成果巩固发展。

（六）生态环境保持良好

实施重点减排项目90个，主要污染物排放得到有效控制，万元GDP能耗进一步下降。环境风险防控措施有效落实，重金属治理项目加快推进，医疗废弃物处置中心投入运营。清理海上违规养殖9890亩、非法围填海1150亩。造林绿化42.4万亩，治理水土流失23.8万亩，矿山复绿行动深入开展。全市主要流域水环境功能区和饮用水源水质达标率达100%，环境空气质量优良天数比例99.2%，创建一批省级以上生态县、生态乡镇和生态村。

（七）政府建设有效加强

自觉接受人大及其常委会监督，依法执行人大及其常委会决议、决定，坚持向人大及其常委会报告工作，积极配合人大常委会开展执法检查、代表视察、专项调研和专题询问，办理市人大代表建议275件，满意率97.1%。支持政协履行政治协商、民主监督、参政议政职能，定期向政协通报情况，积极采纳委员调研成果，办理市政协提案288件，满意率97.9%。广泛听取社会各方面意见。坚持依法行政，政府信息公开、行政复议和行政执法监督有效加强。认真贯彻中央“八项规定”，以“作风转变落实年”为载体，落实“无会周”制度，深入开展“四下基层、四解四促”活动，改进会风文风，提高机关效能。严格落实党风廉政建设责任制，强化行政监察和审计监督，反腐倡廉工作不断深化。

各位代表，成绩的取得来之不易。这是中央和省委、省政府正确领导、关心支持的结果，是全市人民在市委坚强领导下团结一致、艰苦奋斗的结果，是市人大、市政协加强监督、鼎力支持的结果。在此，我代表市人民政府，向为“六新大宁德”建设付出辛勤劳动的全市人民，向给予政府工作有力支持和有效监督的人大代表、政协委员、各民主党派、工商联、各人民团体、无党派人士、离退休老同志和社会各界人士，向为宁德发展作出积极贡献的中央及省属驻宁机构、驻宁部队、武警官兵、公安民警，表示衷心的感谢和崇高的敬意！

在看到成绩的同时，我们也清醒认识到，发展中还面临不少困难和问题，主要是：产业总体实力和竞争力不强，缺乏龙头骨干企业，中小微企业生产经营还面临较大困难；中心城市带动力不强，城乡规划建设管理水平不高，一些地方“两违”现象比较突出；生态保护和节能减排压力较大，教育、医疗等民生事业欠账还比较多，征地拆迁、社会治安、安全生产、食品药品安全等方面还存在不少问题；一些工作人员的责任意识、服务能力、工作效率有待进一步增强，不作为、乱作为和消极腐败等现象仍不同程度存在。我们一定高度重视这些问题，采取更加有力的措施切实加以解决。

二、扎实做好2014年政府工作

今年政府工作的总体要求是：深入贯彻党的十八大、十八届三中全会和习近平总书记系列重要讲话精神，全面落实中央、全省经济工作会议和省委九届九次、十次全会要求，按照市委部署，坚持稳中求进、改革创新，以实施深化“五大战役”重点行动计划为抓手，着力稳增长、调结构，立龙头、抓项目，重扶贫、惠民生，保稳定、促和谐，加快推进“六新大宁德”建设，努力实现“百姓富”与“生态美”的有机统一。

经济社会发展的主要预期目标是：全市生产总值增长12.5%；公共财政总收入增长15%，地方公共财政收入增长17%；全社会固定资产投资增长35%；外贸出口增长20%，实际利用外资增长20%；社会消费品零售总额增长15%，居民消费价格总水平涨幅控制在3.5%以内；城镇居民人均可支配收入增长13%，农民人均纯收入增长15%；城镇登记失业率控制在4%以内；人口自然增长率控制在9.5‰以内；完成单位生产总值能耗、化学需氧量、二氧化硫、氨氮、氮氧化物等年度节能减排任务。

实现上述目标，重点做好八个方面工作：

（一）夯实“三农”基础，提升农业现代化水平

切实保障粮食安全。坚守基本农田红线，有效治理耕地抛荒，补充耕地1.45万亩。实施粮食高产创建行动，建设500亩以上集中连片粮食产能区10个。加快蕉城8万吨国家粮食储备库和市县级现代化仓容建设，新增粮食储备3万吨。拓宽引粮入宁渠道，支持粮油加工、流通企业发展，确保市场供应量足价稳。建立原产地可追溯制度和质量标识制度，持续治理“餐桌污染”，建设“食品放心工程”。

大力发展现代农业。围绕“两带一区”布局，实施现代农业加快发展行动计划，全年完成投资9亿元。提升福建农民创业园、示范基地和市级千亩高优农业示范园建设水平，建设30个以上市级山地农业开发示范基地、6个设施渔业产业园区，新增各类设施农业面积6000亩以上。围绕设施化、集约化、标准化，提升发展茶叶、水产、食用菌三大主导产业，壮大蔬果、畜禽、林竹、花卉、中药材等特色产业。加强种质资源保护和繁育基地建设，培育发展育繁推一体化种业企业。加大农业科技推广力度，推进无土基质栽培等现代技术应用，推广水肥一体化等机械装备。加强防汛抗旱和动植物疫病防控，健全防灾减灾救灾体系。

培育新型经营主体。探索建立更加紧密的利益联结机制，推行“公司+合作社+基地+农户”的经营模式，推广土地托管和经营权入股、信托等土地流转方式，促进适度规模经营。扶持发展农业龙头企业，培育市级以上农民专业合作示范社50家，新增家庭农场100家，培训新型职业农民1000人以上。支持发展专业化服务组织，提高基层农业公共服务能力。扎实推进扶贫开发。坚持开发式扶贫，健全专项扶贫、对口扶贫、社会扶贫机制，加大6个省级扶贫开发重点县、20个市级扶贫开发重点乡镇扶持政策落实力度，实施第四轮整村推进扶贫开发和老区村跨越发展工程，支持少数民族集聚区、海岛、库区等地区加快发展。完善扶贫小额信贷制度，支持贫困户发展种、养、加项目。提高集中安置区规划建设水平，完成“造福工程”危房改造2万人，两年内基本完成已查明地灾点居民搬迁。

（二）加快转型升级，不断增强产业实力

促进工业提质增效。推动信息化与工业化深度融合，实施一批“两化”融合示范项目。抓好100项重点技改项目，完成投资195亿元。引导企业拓展专业化分工协作，提高产品就地配套率，提升产业整体竞争力。实施中小微企业成长行动，建立以税收贡献、技术创新和生态效益为导向的促进机制，扩大企业“成长贷”、“助保贷”业务，做好区域集优票据后续发行工作，鼓励改制企业到海峡股权交易所和“新三板”市场挂牌，实现企业上市新突破。完善工业园区基础设施配套，引导企业“退城入园”，集中力量打造专业化、集约化、规模化的产业集聚区。

做大做强行业龙头。实施产业龙头促进计划，以龙头崛起带动产业链延伸、集群化发展。电机电器产业坚持低成本路线和高附加值路线“两条腿”走路，重点发展具有自主知识产权的高效、超高效电机产品，推动化油器产业升级换代；冶金新材料产业围绕“两片区两延伸”布局，拓展下游精深产品加工链，建设全国不锈钢加工生产基地和贸易中心；能源产业重点加快核电建设，优化风电结构，支持新型动力电池、生物乙醇等新能源发展，打造清洁能源基地；船舶产业大力发展海洋工程船、特种船舶制造，鼓励企业整合重组，培育一批有实力、有竞争力的现代造船龙头企业；医药和医疗器械产业重点加快研发中医药特色产品，引导按摩保健器材企业向医疗器械产业转型；食品加工产业重点加快现代生物、发酵工程等先进技术应用，提升精细加工和市场营销水平；建筑建材产业重点扶持有条件的骨干企业做大做强，支持新型建材企业拓展市场、扩大规模。

强化科技创新驱动。积极争取国家和省级科技重大专项、产业集群项目。支持创建产学研技术创新战略联盟，加快建设企业研发机构，推进科研成果转化和产业化。加快中科高新技术产业示范园、宁德国家农业科技园建设，大力培育科技型企业。加强知识产权创造、运用和保护，完善创业孵化、技术交易、评估咨询等服务。实施“海纳百川”高端人才聚集计划，培养引进一批创新领军人才。

大力发展第三产业。加快旅游产业发展，做好宁德世界地质公园中期评估迎检工作，实施白水洋·鸳鸯溪、太姥山景区提升工程，支持白云山、斗姆岛创建国家4A级景区；有效整合旅游资源，完善旅游基础设施，扩大旅游客源市场，加快旅游“六个一工程”建设，促进观光旅游向休闲度假旅游转型。加快一批重点商贸综合体建设，提升一批茶叶、水产品、食用菌、中药材等专业市场，拓展一批城乡营销网点，积极培育市区中心商圈、县城副中心商圈和社区便民商圈。大力发展道口经济、场站经济，合理布局建设一批综合性物流园区和配送中心。启动促进电子商务发展三年行动，加快重点行业商务平台建设，推动海西药城电子商务平台正式投入运行。引导房地产市场健康平稳发展，满足多样化的住房需求。积极发展会展营销、服务外包、健康服务等新型业态。

加快发展海洋经济。积极培育临港装备制造、海洋生物医药、游艇建造等新兴产业，发展壮大海产品精深加工及冷链物流业。调整海西宁德工业区建设规划，加快开发进程。大力发展远洋渔业，鼓励建设湾外深水抗风浪网箱养殖基地。合理保护和开发岸线资源，科学开展填海造地，加快霞浦福宁湾、蕉城三屿等围垦项目建设，推动福安湖塘、下邳等围垦项目尽快动工。

（三）统筹城乡发展，积极稳妥推进新型城镇化

有序推进农业转移人口市民化。衔接出台具体可操作的户籍制度改革措施，有序放开中心城市、县城和小城镇落户限制，引导和鼓励农业转移人口就近落户城镇，促进有能力在城镇稳定就业和生活的常住人口实现市民化。推动教育、医疗等基本公共服务由户籍人口向常住人口覆盖，加快将符合条件的流动人口纳入城镇住房和社会保障体系。

优化城镇规划布局。以中心城市为龙头，加快中心城区“东扩面海、北展南移”步伐，推进沿海环三都澳城镇群发展，加快建设山区生态城、生态镇，形成“临海布局、环湾发展，山区多点、绿色发展”的城镇化格局。坚持节约集约用地，统筹地上地下空间，提高城镇建设用地利用效率。提升城乡规划设计水平，注重文化传承和生态保护，促进城镇乡村融入自然环境、延续历史文脉，为人民群众留住山水、留住记忆、留住文化和精神的根。

提高城镇承载能力。着力提高中心城市带动力，支持蕉城区打造“产业城·先行区”，鼓励东侨开发区在产业培育、城市建设、项目引进方面创新体制机制、积极先行先试。引导沿海和山区发挥各自比较优势，探索差异化发展道路，努力培育有竞争力的优势产业，打造充满活力的县域经济体和一批各具特色的小城镇。加强城镇综合交通、供水排水、垃圾处理、电网和燃气等基础设施建设，完善商贸流通、教育卫生等功能配套，为城镇人口集中、产业集聚提供有力支撑。

美化净化城乡环境。实施宜居环境建设行动计划，完成年度投资90亿元以上。延伸拓展“点线面”攻坚，提升城区“三边三节点”设计与建设层次，加快中心城区高速桥下整治工程、东湖中央公园、金溪休闲景观带等一批城镇景观建设。深化农村家园清洁行动，力争镇村生活垃圾集中收运率达70%以上，生活污水处理率沿海达40%以上、山区达30%以上，整治村庄130个，创建10个美丽乡村示范村。

加强城镇综合管理。创新城镇建设投融资机制，做大政府性融资平台，鼓励社会资本参与投资运营城镇公共设施。健全“大城管”机制，进一步完善中心城区市、区两级行政执法体制，所有县（市、区）建成数字城管系统。健全完善整治环境卫生、交通秩序、占道经营的长效机制。深入开展“两违”专项治理行动，坚决遏制城乡“两违”蔓延势头。

（四）扩大有效投资，进一步增强发展后劲

提高“三维”对接层次。健全“三维”项目跟踪管理信息系统，完善项目储备库动态管理机制。坚持招商选商，注重项目用地投资强度、税收贡献和节能环保要求，提升“三维”对接质量。实施园区招商、以商招商，围绕冶金、能源、电机等优势主导产业，突出对接一批产业龙头项目和产业链关键配套项目，力争全年签约“三维”项目总投资1200亿元，上年签约项目开工率80%以上，当年签约项目开工率50%以上。

全力实施重点项目。年度计划安排重点项目370个，完成在建重点项目年度投资480亿元。继续集中力量推进“双百项目”建设，完成年度投资350亿元。深化项目前期工作，强化项目业主责任制，提高可研深度，推动项目早开工、早建设。落实重点项目建设“路线图”，完善项目协调调度、跟踪服务和督查考评机制，全面推行施工标准化管理。

强化基础设施建设。实施城乡基础设施提升行动计划，全年建设项目239个、完成投资突破300亿元。提升交通路网，力争动工建设衢宁铁路宁德段，加快建设合福铁路宁德段和京台高速宁德段等4条（段）高速公路，开工建设沈海复线福安至漳湾段等3条（段）高速公路，建设普通公路干线341公里，建成农村路网180公里，加快建设三都澳航道一期工程等12个港航项目，续建新建一批中心渔港和一、二级渔港，实施海岛交通便民工程。强化能源保障，实现宁德核电2号机组商业运行，开工建设宁德核电二期工程5号、6号机组，新建扩建各级变电站13座、110千伏及以上线路180.7公里。完善市政设施，建成闽东中路、富春东路等一批城市主干道，加快推进滨海大道及内侧围填海前期工作，继续抓好一批停车场、公厕等市政设施建设，全市新建改建绿道和城区雨污管网、供水管网、燃气管网、城市道路各100公里。加快水利建设，续建官昌水库、赛江防洪二期工程等10个重大水利项目，加快上白石水利枢纽、吴坑水库等8个重大水利项目前期工作，推进小型农田水利县建设，继续抓好海堤、水库除险加固和中小河流治理，解决17万农村居民饮水安全问题。

（五）突出文化引领，丰富人民群众精神生活

加强精神文明建设。积极倡导和践行社会主义核心价值观，大力弘扬“滴水穿石”的闽东精神，举办闽东苏区创建80周年纪念活动。加强公民道德建设和未成年人思想道德教育，实施市民素质教育工程，深化群众性精神文明创建活动，努力争创省级文明城市。

繁荣发展文化事业。实施重点文化惠民工程，持续扩大“魅力闽东”文化惠民品牌影响，启动建设市体育馆、博物馆和档案馆。深入挖掘闽东特色文化内涵，鼓励创作文化精品。重视文化遗产、历史文化名镇（村）保护和开发，加快闽东北木拱廊桥申报世界文化遗产步伐，推进第二轮地方志修编。广泛开展全民健身活动，认真筹备第一届全国青运会击剑比赛，积极备战第十五届省运会。

培育壮大文化产业。实施文化产业“七个一”工程，加快建设宁德工艺博览城、霞浦国际滨海影视文化创意产业园，扶持畲族银器、柘荣剪纸、霞浦木石盆景、蕉城仿古家具、寿宁乌金陶艺、古田双坑油画等特色文化产业示范基地做大做优，培育一批文化产业龙头企业。推进文化与旅游深度融合发展，加快建设一批重点文化旅游项目。

（六）坚持民生优先，着力提高公共服务能力

努力增加居民收入。完善公共就业创业服务体系，统筹做好以大中专毕业生为重点的青年就业和农村转移劳动力、城镇就业困难人员、退役军人就业工作，全年城镇新增就业2.8万人、农村劳动力转移就业4万人。推进企业工资集体协商，健全工资正常增长机制，深化“创建无欠薪项目部”活动，确保农民工工资及时足额兑现。完善农村征地补偿机制，稳妥开展农民住房财产权抵押、担保、转让试点，探索农民增加财产性收入渠道。

健全社会保障体系。实施民生工程行动计划，办好39件为民办实事项目。完善城乡居民社会养老保险一体化制度，实现养老保险人员全覆盖，稳步提高养老保险待遇水平。推进城乡居民医疗保险一体化，提高基本医疗保险财政补助标准。健全价格监测预警体系，完善社会救助和保障标准与食品价格上涨挂钩联动机制。抓好保障性安居工程建设，改造各类棚户区4180户、新建保障性住房4225套。提高城乡低保标准和“五老”、“五保户”供养水平，健全临时救助制度。做好关心下一代和家庭困难学生资助工作，关注农村留守老人妇女儿童，支持社会力量举办养老机构，重视发展社会福利、慈善、老龄、红十字和残疾人事业。

办好人民满意教育。实施教育扶贫攻坚计划，支持名校办分校、老校带新校、强校扶弱校，推进优质教育资源共建共享。全面完成义务教育学校标准化建设，加快实施宁师附小、宁德五中和福安一中新校区等一批城区中小学校扩容工程，新增城区学位3000个。启动新一轮学前教育三年行动计划。推动普通高中达标晋级。深化职业教育产教融合、校企合作。继续推进宁德师范学院新校区三期工程、宁德职业技术学院二期工程建设，筹建宁德卫生职业学院。扶持和规范民办教育发展，办好继续教育、特殊教育、老年教育、民族教育。启动实施“名师名校长培养工程”，加强师德师风建设，提高教师队伍整体素质。

加快发展卫生计生事业。进一步扩充和优化配置医疗卫生资源，全年新增医疗机构床位400张以上。深化基层医疗卫生机构综合改革，完善基本药物制度，促进基本药物优先合理配备使用。稳步推进公立医院改革，探索建立多种形式的医疗联合体。推进市级三甲医院重点专科和市妇幼保健院建设，提升县级医院服务能力。加强乡村卫生机构建设，全面推广“海云工程”，提升基层中医药服务能力。大力支持社会资本办医，优先支持举办非营利性医疗机构。深入开展爱国卫生运动，完善疾病预防控制体系，提高突发公共卫生事件应急处置能力。衔接落实“单独二孩”政策，综合治理出生人口性别比偏高问题，提高出生人口素质。

推进社会治理创新。深化“平安宁德”建设，强化社会治安动态管控。健全重大决策社会稳定风险评估和应急处置机制，严格按照“路线图”依法处理信访事项，积极化解信访积案。完善社区网格化服务管理，做好普法教育、法律援助等工作。健全安全生产责任体系，加强重点行业领域隐患排查治理和重大危险源监控，继续推进道路交通安全综合整治和“清剿火患”专项行动，有效防范重大安全生产事故。深化食品药品安全专项整治，加大生产、流通、消费等环节监管力度，全力保障群众饮食用药安全。培育和发展社会组织，支持工会、共青团、妇联等人民团体发挥桥梁纽带作用。加强国防动员、民兵预备役、双拥共建和海防、人防、打私等工作。大力促进民族团结，依法管理宗教事务。

（七）促进生态文明，加快建设“美丽宁德”

深化生态示范创建。加快创建国家级生态市，力争实现省级生态市创建目标，国家级生态乡镇创建率80%以上，支持柘荣县建成国家级生态县，中心城市和福鼎市、福安市建成国家环保模范城市。加快“四绿工程”建设，实施造林绿化16.4万亩，治理水土流失18.4万亩。加强自然保护区规划、建设和管理，推进湿地保护，深化海上养殖综合整治，开展矿山生态恢复治理。研究建立生态补偿机制，科学划定生态红线，完善环保监管“一岗双责”。

积极促进节能减排。加强单位生产总值能耗和能源消费总量双控管理，严格执行空间、总量、项目“三位一体”环境准入机制，推动建立排污权有偿使用和交易制度，完善节能减排指标、监测和考核三大体系。发展循环经济，推行清洁生产，加快淘汰落后产能，鼓励重点用能企业开展节能低碳行动。建立水资源利用总量、用水效率、水功能区限制纳污控制指标体系，推进节水示范工程。

持续改善环境质量。深化水污染治理，继续推进“两江三溪”重点流域水环境综合整治，加大饮用水源地环境保护力度，加强规模化畜禽养殖场治理，新建、扩建城镇生活污水处理厂12座，推进工业园区污水集中治理设施建设。深化大气污染防治，提高火电、冶金等企业脱硫、脱硝效率，推进合成革企业清洁能源替代工程，落实非绿标车限行措施，整治建筑施工、道路扬尘和饮食业油烟污染。健全环境执法联动机制，建设一批水、空气、辐射等环境质量自动监测站，开展PM2.5检测，强化重金属、核与辐射、危险废物环境监管，提高防范环境风险能力。

（八）深化改革开放，充分释放发展活力动力

推进重点领域改革。深化行政体制改革，全面完成市县政府机构改革任务，稳步推进事业单位分类改革。深化行政审批制度改革，进一步下放审批权限，力争“十二五”末在全省率先基本取消市级项目审批事项。推进工商登记制度改革，放宽经营范围登记，试行“先照后证”登记制。大力发展民营经济，引导社会资本参与交通、能源、市政、水利、通信等基础产业领域的建设和运营管理。推动国资监管体制改革，完善财政统筹、国资监控的融资平台，进一步整合市属国企。认真做好第三次全国经济普查工作。深化农村综合改革，完善乡镇产权交易平台，鼓励林权和土地规范流转，推进农村土地确权登记发证。推动金融领域改革，加强金融风险防控，大力推进“引银入宁”，争取厦门银行、民生银行、平安银行在宁德设立分行，规范和引导担保公司转型升级，在全省率先实现村镇银行县域全覆盖。

提升对外开放水平。积极推动三都澳、沙埕港水域对外开放，力争取得新进展。加快建设福安白马港口岸查验园区，试行“多点报检”模式，推进通关便利化。提升完善机电、船舶、水产、茶叶等公共服务平台，支持企业参加国内外重要展会，鼓励企业到境外设立营销服务网点。引导企业创建自主出口品牌，提高产品附加值和竞争力，推进外贸转型升级。拓宽利用外资领域，鼓励外资企业增资扩股，引导外商投资商贸物流、融资租赁、育幼养老等服务业。深化宁港澳侨合作，拓展友城合作领域。

加强宁台交流合作。积极推进宁台产业深度对接，加强与台湾百大企业、高新技术企业和民间商会协会合作，启动建设联德冶金新材料二期项目，促进三沙、沙埕对台小额贸易发展，推动宁台旅游、交通、电子商务等服务领域的对接协作。加强宁台文化交流和人员往来，促进戏曲、工艺、美术等民俗文化和宗亲交流，深入开展青少年往来互动交流活动。办好第五届海峡两岸电博会、第六届海峡论坛·陈靖姑文化节。

建设先行先试平台。积极对接比照享受原中央苏区有关政策，扎实推进申办全国军民融合深度发展试验区工作。科学高效利用山海资源，争取列入全国陆海统筹发展试点城市，启动实施山海资源基础性专项调查，调整修改土地利用总体规划，优化山海国土资源开发利用空间。

三、建设为民务实清廉政府

坚持依法行政，彰显公信力。依法接受人大及其常委会的工作监督和法律监督，主动接受政协民主监督，认真听取各民主党派、工商联、人民团体和无党派人士意见，提高人大代表建议、意见和政协提案办理质量。规范行政决策程序，完善规范性文件和重大决策合法性审查机制，健全政务公开制度，推行政府信息发布和民意反馈机制，做好行政复议和行政应诉工作。

坚持转变作风，提高执行力。按照“照镜子、正衣冠、洗洗澡、治治病”的要求，扎实开展党的群众路线教育实践活动，完善“四下基层、四解四促”制度，着力整治“四风”问题。精简各类检查评比，切实减轻基层负担。贯彻落实机关效能建设条例，拓展政府绩效管理，大力推行“马上就办”，下大气力改进会风文风，以“钉钉子”的精神集中精力抓落实。

坚持廉洁从政，增强约束力。扎实推进惩防体系建设，落实党风廉政建设责任制。认真贯彻中央“八项规定”精神，严格执行党政机关厉行节约反对浪费条例，强化预算约束，严控“三公”经费。坚持用制度管权管事管人，加强政府层级监督和专门监督，高度重视社会公众监督和舆论监督，让权力始终在阳光下运行，做到干部清正、政府清廉、政治清明。

各位代表！新的征程已经开启，新的目标催人奋进。让我们紧密团结在以习近平同志为总书记的党中央周围，在省委、省政府和市委的坚强领导下，紧紧依靠全市人民，改革创新，奋发进取，为开创“六新大宁德”建设新局面、全面建成小康社会而努力奋斗！
